# Ethyl (*E*)-1-(2-styryl-1*H*-benzimidazol-1-yl)acetate

**DOI:** 10.1107/S1600536809019825

**Published:** 2009-06-10

**Authors:** Xue-qun Fu, Guang-hai Xu

**Affiliations:** a Ordered Matter Science Research Center, Southeast UniVersity, Nanjing 210096, People’s Republic of China

## Abstract

In the title compound, C_19_H_18_NO_2_, the dihedral angle between the benzimidazole and phenyl ring planes is 18.18 (17)°. The atoms of the ethyl side chain are disordered over two sets of sites in a 0.50:0.50 ratio. In the crystal, inter­molecular C—H⋯O hydrogen bonds and C—H⋯π contacts help to consolidate the packing.

## Related literature

For further synthetic details, see: Hang & Ye (2008 ▶). For background on benzimidazoles, see: Göker *et al.* (1999 ▶); Özbey *et al.* (1998 ▶).
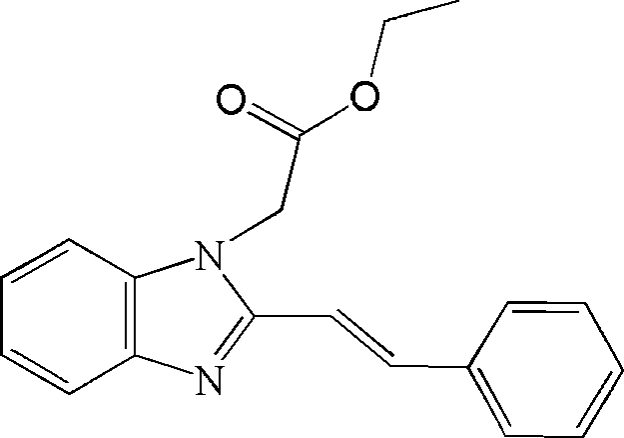


## Experimental

### 

#### Crystal data

C_19_H_18_N_2_O_2_
*M* *_r_* = 307.36
Orthorhombic, 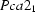

*a* = 12.021 (2) Å
*b* = 14.369 (3) Å
*c* = 9.7517 (18) Å
*V* = 1684.4 (5) Å^3^
*Z* = 4
Mo *K*α radiation
μ = 0.08 mm^−1^
*T* = 298 (2) K
0.25 × 0.25 × 0.20 mm

#### Data collection

Rigaku SCXmini diffractometer
Absorption correction: multi-scan (*CrystalClear*; Rigaku, 2005) *T*_min_ = 0.884, *T*_max_ = 0.984
16640 measured reflections
2046 independent reflections
1545 reflections with *I* > 2σ(*I*)
*R*_int_ = 0.056

#### Refinement

*R*[*F* ^2^ > 2σ(*F* ^2^)] = 0.058
*wR*(*F* ^2^) = 0.151
*S* = 1.07
2046 reflections
215 parameters
43 restraints
H-atom parameters constrained
Δρ_max_ = 0.16 e Å^−3^
Δρ_min_ = −0.25 e Å^−3^

### 

Data collection: *CrystalClear* (Rigaku, 2005 ▶); cell refinement: *CrystalClear*; data reduction: *CrystalClear*; program(s) used to solve structure: *SHELXS97* (Sheldrick, 2008 ▶); program(s) used to refine structure: *SHELXL97* (Sheldrick, 2008 ▶); molecular graphics: *SHELXTL* (Sheldrick, 2008 ▶); software used to prepare material for publication: *SHELXTL*.

## Supplementary Material

Crystal structure: contains datablocks I, global. DOI: 10.1107/S1600536809019825/hb2982sup1.cif

Structure factors: contains datablocks I. DOI: 10.1107/S1600536809019825/hb2982Isup2.hkl

Additional supplementary materials:  crystallographic information; 3D view; checkCIF report

## Figures and Tables

**Table 1:** Hydrogen-bond geometry (Å, °)

*D*—H⋯*A*	*D*—H	H⋯*A*	*D*⋯*A*	*D*—H⋯*A*
C4—H4*B*⋯O1^i^	0.97	2.47	3.409 (4)	162
C4—H4*A*⋯*Cg*2^ii^	0.97	2.67	3.577 (4)	156

Symmetry codes: (i) 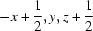; (ii) 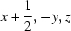; (iii) 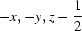.

**Fig. 1.**
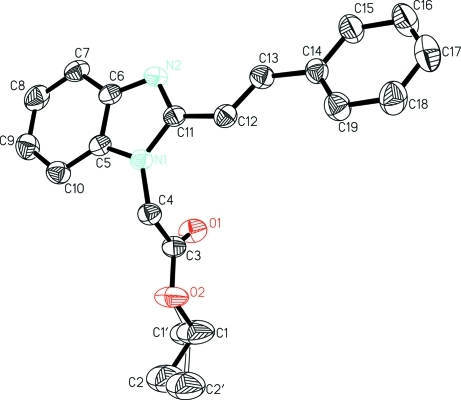
The molecular structure of (I) with displacement ellipsoids drawn at the 30% probability level (all H atoms have been omitted for clarity).

**Fig. 2.**
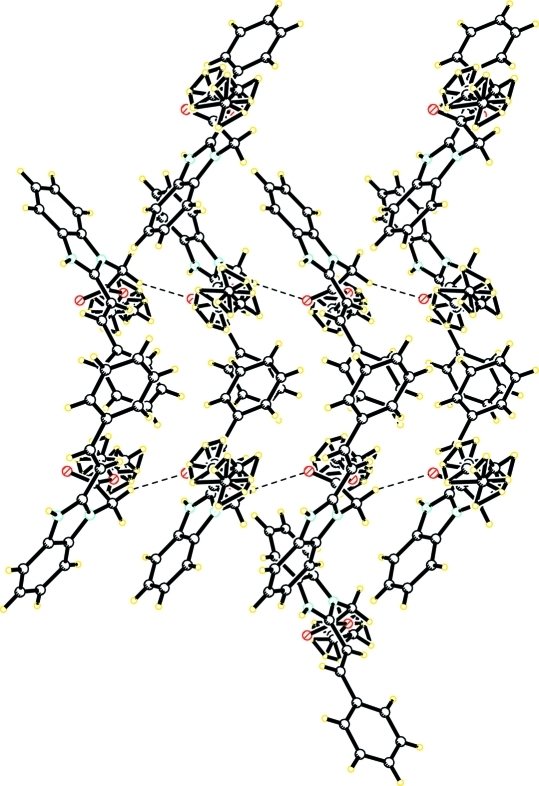
A view of the packing of the title compound, stacking along the b axis. Dashed lines indicate hydrogen bonds.

## supplementary crystallographic information

### Comment

The benzimidazole ring system is of great interest because of its diverse biological activities while the synthesis and crystal structure analyses of several benzimidazoles have already been reported (Göker *et al.*, 1999; Özbey *et al.*, 1998).

In the structure of the title compound (Fig. 1), the benzimidazole system is essentially planar (dihedral angle 1.17 (2)°). The dihedral angle between the benzimidazole and styryl groups is 17.78 (1)°. The molecule is twisted with the N1—C4—C3—O1 torsion angle of 13.61 (4)° between the ethyl acetate and benzimidazole groups.

In the crystal, intermolecular C—H···O hydrogen bonds (Fig.2) link the molecules to chains along the *b* axis. In addition the C—H···π contacts (Table 1) further stabilize the crystal structure.

### Experimental

The synthesis of (*E*)-2-styryl-1*H*-benzimidazole was reported previously (Hang & Ye, 2008). Ethyl 2-bromoacetate (1.65 g. 10 mmol) was added to a solution of (*E*)-2-styryl-1*H*-benzo[*d*]imidazole (2.2 g,10 mmol) and NaH (0.6 g, 26 mmol) in THF (30 ml). After the mixture was stirred for 12 h at room temperature, the precipitate was filtered off and the solution was evaporated in vacuum. The crude product was then crystallized from ethanol to yield colourless prisms of (I).

### Refinement

Anomalous dispersion was negligible and Friedel pairs were merged before refimenent. The positional parameters of all the H atoms were calculated geometrically and refined as riding with *U*_iso_(H) = 1.2*U*_eq_(C,N) or 1.5U_eq_(methyl C).

### Crystal data

**Table:**

C_19_H_18_N_2_O_2_	*F*(000) = 652
*M**_r_* = 307.36	*D*_x_ = 1.212 Mg m^−^^3^
Orthorhombic, *P**c**a*2_1_	Mo *K*α radiation, λ = 0.71073 Å
Hall symbol: P 2c -2ac	Cell parameters from 3237 reflections
*a* = 12.021 (2) Å	θ = 2.5–27.5°
*b* = 14.369 (3) Å	µ = 0.08 mm^−^^1^
*c* = 9.7517 (18) Å	*T* = 298 K
*V* = 1684.4 (5) Å^3^	Prism, colourless
*Z* = 4	0.25 × 0.25 × 0.20 mm

### Data collection

**Table:**

Rigaku SCXmini diffractometer	2046 independent reflections
Radiation source: fine-focus sealed tube	1545 reflections with *I* > 2σ(*I*)
graphite	*R*_int_ = 0.056
Detector resolution: 13.6612 pixels mm^-1^	θ_max_ = 27.5°, θ_min_ = 2.8°
CCD_Profile_fitting scans	*h* = −15→15
Absorption correction: multi-scan (*CrystalClear*; Rigaku, 2005)	*k* = −18→18
*T*_min_ = 0.884, *T*_max_ = 0.984	*l* = −12→12
16640 measured reflections	

### Refinement

**Table:**

Refinement on *F*^2^	Primary atom site location: structure-invariant direct methods
Least-squares matrix: full	Secondary atom site location: difference Fourier map
*R*[*F*^2^ > 2σ(*F*^2^)] = 0.058	Hydrogen site location: inferred from neighbouring sites
*wR*(*F*^2^) = 0.151	H-atom parameters constrained
*S* = 1.07	*w* = 1/[σ^2^(*F*_o_^2^) + (0.0802*P*)^2^ + 0.1398*P*] where *P* = (*F*_o_^2^ + 2*F*_c_^2^)/3
2046 reflections	
215 parameters	Δρ_max_ = 0.16 e Å^−^^3^
43 restraints	Δρ_min_ = −0.25 e Å^−^^3^

### Special details

**Table:**

Geometry. All esds (except the esd in the dihedral angle between two l.s. planes) are estimated using the full covariance matrix. The cell esds are taken into account individually in the estimation of esds in distances, angles and torsion angles; correlations between esds in cell parameters are only used when they are defined by crystal symmetry. An approximate (isotropic) treatment of cell esds is used for estimating esds involving l.s. planes.
Refinement. Refinement of F^2^ against ALL reflections. The weighted R-factor wR and goodness of fit S are based on F^2^, conventional R-factors R are based on F, with F set to zero for negative F^2^. The threshold expression of F^2^ > 2sigma(F^2^) is used only for calculating R-factors(gt) etc. and is not relevant to the choice of reflections for refinement. R-factors based on F^2^ are statistically about twice as large as those based on F, and R- factors based on ALL data will be even larger.

### Fractional atomic coordinates and isotropic or equivalent isotropic displacement parameters (Å^2^)

**Table:**

	*x*	*y*	*z*	*U*_iso_*/*U*_eq_	Occ. (<1)
N1	0.1633 (2)	0.13879 (18)	0.1323 (3)	0.0509 (6)	
N2	0.0004 (2)	0.14329 (18)	0.0204 (3)	0.0558 (7)	
C5	0.1630 (2)	0.0626 (2)	0.0454 (3)	0.0500 (7)	
O1	0.3290 (2)	0.2529 (2)	0.0215 (3)	0.0743 (7)	
O2	0.4352 (2)	0.2271 (2)	0.2042 (3)	0.0925 (10)	
C3	0.3424 (3)	0.2197 (2)	0.1332 (4)	0.0570 (8)	
C6	0.0611 (3)	0.0668 (2)	−0.0229 (4)	0.0543 (8)	
C11	0.0644 (2)	0.1847 (2)	0.1111 (3)	0.0493 (7)	
C12	0.0354 (3)	0.2693 (2)	0.1853 (4)	0.0559 (8)	
H12A	0.0799	0.2873	0.2586	0.067*	
C13	−0.0510 (3)	0.3216 (2)	0.1534 (4)	0.0587 (8)	
H13A	−0.0924	0.3024	0.0780	0.070*	
C14	−0.0894 (3)	0.4059 (2)	0.2221 (4)	0.0586 (8)	
C4	0.2583 (3)	0.1664 (2)	0.2144 (3)	0.0539 (7)	
H4A	0.2932	0.1113	0.2524	0.065*	
H4B	0.2329	0.2046	0.2903	0.065*	
C7	0.0364 (3)	−0.0019 (3)	−0.1204 (4)	0.0675 (10)	
H7A	−0.0309	−0.0015	−0.1675	0.081*	
C15	−0.1778 (4)	0.4562 (3)	0.1668 (5)	0.0794 (12)	
H15A	−0.2105	0.4360	0.0856	0.095*	
C19	−0.0430 (3)	0.4385 (3)	0.3420 (4)	0.0715 (10)	
H19A	0.0171	0.4072	0.3806	0.086*	
C9	0.2151 (4)	−0.0720 (3)	−0.0755 (4)	0.0763 (11)	
H9A	0.2658	−0.1190	−0.0953	0.092*	
C18	−0.0843 (4)	0.5169 (3)	0.4058 (5)	0.0863 (13)	
H18A	−0.0519	0.5378	0.4867	0.104*	
C8	0.1142 (4)	−0.0697 (3)	−0.1441 (5)	0.0804 (12)	
H8A	0.0987	−0.1157	−0.2085	0.096*	
C10	0.2420 (3)	−0.0058 (2)	0.0215 (4)	0.0652 (9)	
H10A	0.3094	−0.0070	0.0684	0.078*	
C16	−0.2178 (4)	0.5352 (3)	0.2300 (6)	0.0944 (14)	
H16A	−0.2757	0.5686	0.1902	0.113*	
C17	−0.1726 (5)	0.5644 (3)	0.3512 (6)	0.0941 (15)	
H17A	−0.2015	0.6161	0.3962	0.113*	
C1	0.5262 (14)	0.283 (2)	0.143 (3)	0.128 (3)	0.50
H1A	0.5213	0.2820	0.0437	0.153*	0.50
H1B	0.5218	0.3470	0.1738	0.153*	0.50
C2	0.628 (3)	0.241 (2)	0.188 (3)	0.137 (8)	0.50
H2B	0.6899	0.2732	0.1468	0.206*	0.50
H2C	0.6296	0.1769	0.1606	0.206*	0.50
H2D	0.6336	0.2453	0.2858	0.206*	0.50
C1'	0.5333 (14)	0.271 (2)	0.137 (3)	0.128 (3)	0.50
H1'A	0.5564	0.2338	0.0590	0.153*	0.50
H1'B	0.5147	0.3327	0.1058	0.153*	0.50
C2'	0.621 (3)	0.275 (2)	0.237 (3)	0.137 (8)	0.50
H2'A	0.6839	0.3070	0.1982	0.206*	0.50
H2'B	0.6429	0.2130	0.2622	0.206*	0.50
H2'C	0.5958	0.3078	0.3167	0.206*	0.50

### Atomic displacement parameters (Å^2^)

**Table:**

	*U*^11^	*U*^22^	*U*^33^	*U*^12^	*U*^13^	*U*^23^
N1	0.0473 (13)	0.0574 (15)	0.0480 (13)	−0.0008 (12)	−0.0054 (12)	−0.0040 (13)
N2	0.0486 (13)	0.0628 (16)	0.0560 (15)	−0.0030 (13)	−0.0017 (13)	−0.0031 (15)
C5	0.0513 (17)	0.0551 (17)	0.0434 (17)	−0.0044 (15)	0.0014 (14)	−0.0030 (14)
O1	0.0745 (16)	0.0925 (18)	0.0560 (14)	−0.0139 (14)	−0.0052 (14)	0.0121 (15)
O2	0.0618 (15)	0.125 (2)	0.090 (2)	−0.0270 (16)	−0.0254 (16)	0.043 (2)
C3	0.0547 (18)	0.0639 (19)	0.0525 (19)	−0.0027 (16)	−0.0054 (15)	−0.0003 (17)
C6	0.0513 (18)	0.0607 (18)	0.0510 (17)	−0.0093 (15)	0.0032 (14)	−0.0006 (16)
C11	0.0451 (16)	0.0533 (16)	0.0495 (17)	−0.0047 (14)	0.0063 (14)	0.0020 (15)
C12	0.0571 (19)	0.0557 (18)	0.0550 (19)	−0.0045 (16)	0.0044 (15)	0.0012 (16)
C13	0.0571 (19)	0.0631 (19)	0.0560 (19)	0.0008 (16)	−0.0026 (15)	−0.0011 (17)
C14	0.0614 (19)	0.0538 (17)	0.0604 (19)	0.0014 (16)	0.0056 (18)	0.0043 (17)
C4	0.0559 (18)	0.0615 (16)	0.0442 (15)	−0.0003 (17)	−0.0074 (15)	0.0007 (16)
C7	0.060 (2)	0.074 (2)	0.068 (2)	−0.0138 (19)	−0.0028 (18)	−0.015 (2)
C15	0.082 (3)	0.071 (2)	0.085 (3)	0.016 (2)	−0.010 (2)	−0.005 (2)
C19	0.075 (2)	0.073 (2)	0.067 (2)	0.0104 (19)	0.0006 (19)	−0.005 (2)
C9	0.082 (3)	0.068 (2)	0.079 (2)	0.010 (2)	0.002 (2)	−0.017 (2)
C18	0.105 (3)	0.080 (3)	0.074 (3)	0.006 (3)	−0.003 (3)	−0.016 (2)
C8	0.089 (3)	0.072 (2)	0.080 (3)	−0.010 (2)	0.000 (2)	−0.027 (2)
C10	0.0597 (18)	0.0716 (19)	0.064 (2)	0.0050 (18)	−0.0025 (18)	−0.0081 (19)
C16	0.100 (4)	0.082 (3)	0.101 (3)	0.034 (3)	−0.008 (3)	−0.003 (3)
C17	0.112 (4)	0.074 (3)	0.096 (3)	0.025 (3)	0.022 (3)	−0.004 (3)
C1	0.078 (3)	0.160 (7)	0.145 (6)	−0.048 (4)	−0.024 (4)	0.068 (6)
C2	0.086 (5)	0.18 (2)	0.151 (19)	−0.025 (9)	−0.005 (10)	0.031 (12)
C1'	0.078 (3)	0.160 (7)	0.145 (6)	−0.048 (4)	−0.024 (4)	0.068 (6)
C2'	0.086 (5)	0.18 (2)	0.151 (19)	−0.025 (9)	−0.005 (10)	0.031 (12)

### Geometric parameters (Å, °)

**Table:**

N1—C11	1.376 (4)	C15—H15A	0.9300
N1—C5	1.384 (4)	C19—C18	1.380 (6)
N1—C4	1.450 (4)	C19—H19A	0.9300
N2—C11	1.314 (4)	C9—C10	1.380 (5)
N2—C6	1.385 (4)	C9—C8	1.387 (6)
C5—C10	1.387 (5)	C9—H9A	0.9300
C5—C6	1.395 (4)	C18—C17	1.369 (7)
O1—C3	1.200 (5)	C18—H18A	0.9300
O2—C3	1.318 (4)	C8—H8A	0.9300
O2—C1	1.482 (9)	C10—H10A	0.9300
O2—C1'	1.485 (9)	C16—C17	1.367 (8)
C3—C4	1.495 (5)	C16—H16A	0.9300
C6—C7	1.402 (5)	C17—H17A	0.9300
C11—C12	1.457 (5)	C1—C2	1.434 (10)
C12—C13	1.318 (5)	C1—H1A	0.9700
C12—H12A	0.9300	C1—H1B	0.9700
C13—C14	1.459 (5)	C2—H2B	0.9600
C13—H13A	0.9300	C2—H2C	0.9600
C14—C19	1.378 (5)	C2—H2D	0.9600
C14—C15	1.393 (5)	C1'—C2'	1.436 (10)
C4—H4A	0.9700	C1'—H1'A	0.9700
C4—H4B	0.9700	C1'—H1'B	0.9700
C7—C8	1.370 (6)	C2'—H2'A	0.9600
C7—H7A	0.9300	C2'—H2'B	0.9600
C15—C16	1.378 (6)	C2'—H2'C	0.9600
			
C11—N1—C5	106.6 (2)	C10—C9—C8	121.3 (4)
C11—N1—C4	129.2 (3)	C10—C9—H9A	119.3
C5—N1—C4	123.8 (3)	C8—C9—H9A	119.3
C11—N2—C6	104.9 (3)	C17—C18—C19	120.7 (5)
N1—C5—C10	131.4 (3)	C17—C18—H18A	119.6
N1—C5—C6	105.1 (3)	C19—C18—H18A	119.6
C10—C5—C6	123.5 (3)	C7—C8—C9	122.2 (4)
C3—O2—C1	117.2 (11)	C7—C8—H8A	118.9
C3—O2—C1'	118.4 (10)	C9—C8—H8A	118.9
C1—O2—C1'	8(3)	C9—C10—C5	116.3 (4)
O1—C3—O2	124.0 (3)	C9—C10—H10A	121.8
O1—C3—C4	126.4 (3)	C5—C10—H10A	121.8
O2—C3—C4	109.6 (3)	C17—C16—C15	120.0 (5)
N2—C6—C5	110.6 (3)	C17—C16—H16A	120.0
N2—C6—C7	130.8 (3)	C15—C16—H16A	120.0
C5—C6—C7	118.7 (3)	C16—C17—C18	119.4 (4)
N2—C11—N1	112.9 (3)	C16—C17—H17A	120.3
N2—C11—C12	124.9 (3)	C18—C17—H17A	120.3
N1—C11—C12	122.2 (3)	C2—C1—O2	106 (2)
C13—C12—C11	123.1 (3)	C2—C1—H1A	110.5
C13—C12—H12A	118.4	O2—C1—H1A	110.5
C11—C12—H12A	118.4	C2—C1—H1B	110.5
C12—C13—C14	127.9 (3)	O2—C1—H1B	110.5
C12—C13—H13A	116.1	H1A—C1—H1B	108.6
C14—C13—H13A	116.1	C1—C2—H2B	109.5
C19—C14—C15	117.4 (4)	C1—C2—H2C	109.5
C19—C14—C13	122.9 (3)	H2B—C2—H2C	109.5
C15—C14—C13	119.6 (3)	C1—C2—H2D	109.5
N1—C4—C3	112.3 (3)	H2B—C2—H2D	109.5
N1—C4—H4A	109.1	H2C—C2—H2D	109.5
C3—C4—H4A	109.1	C2'—C1'—O2	108 (2)
N1—C4—H4B	109.1	C2'—C1'—H1'A	110.1
C3—C4—H4B	109.1	O2—C1'—H1'A	110.1
H4A—C4—H4B	107.9	C2'—C1'—H1'B	110.1
C8—C7—C6	118.0 (4)	O2—C1'—H1'B	110.1
C8—C7—H7A	121.0	H1'A—C1'—H1'B	108.4
C6—C7—H7A	121.0	C1'—C2'—H2'A	109.5
C16—C15—C14	121.4 (5)	C1'—C2'—H2'B	109.5
C16—C15—H15A	119.3	H2'A—C2'—H2'B	109.5
C14—C15—H15A	119.3	C1'—C2'—H2'C	109.5
C14—C19—C18	121.0 (4)	H2'A—C2'—H2'C	109.5
C14—C19—H19A	119.5	H2'B—C2'—H2'C	109.5
C18—C19—H19A	119.5		
			
C11—N1—C5—C10	178.2 (4)	C12—C13—C14—C15	174.9 (4)
C4—N1—C5—C10	4.8 (5)	C11—N1—C4—C3	−91.5 (4)
C11—N1—C5—C6	−0.8 (3)	C5—N1—C4—C3	80.3 (4)
C4—N1—C5—C6	−174.2 (3)	O1—C3—C4—N1	13.5 (5)
C1—O2—C3—O1	2.3 (17)	O2—C3—C4—N1	−167.8 (3)
C1'—O2—C3—O1	−6.5 (17)	N2—C6—C7—C8	178.9 (4)
C1—O2—C3—C4	−176.4 (16)	C5—C6—C7—C8	−0.3 (5)
C1'—O2—C3—C4	174.8 (16)	C19—C14—C15—C16	−0.4 (6)
C11—N2—C6—C5	0.7 (4)	C13—C14—C15—C16	178.8 (4)
C11—N2—C6—C7	−178.6 (4)	C15—C14—C19—C18	1.2 (6)
N1—C5—C6—N2	0.1 (3)	C13—C14—C19—C18	−177.9 (4)
C10—C5—C6—N2	−179.0 (3)	C14—C19—C18—C17	0.0 (7)
N1—C5—C6—C7	179.5 (3)	C6—C7—C8—C9	−0.1 (7)
C10—C5—C6—C7	0.4 (5)	C10—C9—C8—C7	0.4 (7)
C6—N2—C11—N1	−1.2 (4)	C8—C9—C10—C5	−0.3 (6)
C6—N2—C11—C12	180.0 (3)	N1—C5—C10—C9	−178.9 (3)
C5—N1—C11—N2	1.3 (4)	C6—C5—C10—C9	−0.1 (5)
C4—N1—C11—N2	174.2 (3)	C14—C15—C16—C17	−1.7 (8)
C5—N1—C11—C12	−179.8 (3)	C15—C16—C17—C18	2.9 (8)
C4—N1—C11—C12	−7.0 (5)	C19—C18—C17—C16	−2.0 (8)
N2—C11—C12—C13	−11.1 (5)	C3—O2—C1—C2	−147.8 (16)
N1—C11—C12—C13	170.2 (3)	C1'—O2—C1—C2	−47 (14)
C11—C12—C13—C14	178.3 (3)	C3—O2—C1'—C2'	177.1 (14)
C12—C13—C14—C19	−5.9 (6)	C1—O2—C1'—C2'	94 (16)

### Hydrogen-bond geometry (Å, °)

**Table:**

*D*—H···*A*	*D*—H	H···*A*	*D*···*A*	*D*—H···*A*
C4—H4B···O1^i^	0.97	2.47	3.409 (4)	162
C4—H4A···Cg2^ii^	0.97	2.67	3.577 (4)	156

Symmetry codes: (i) −*x*+1/2, *y*, *z*+1/2; (ii) *x*+1/2, −*y*, *z*.
